# Kinetics and Ligand-Binding Preferences of *Mycobacterium tuberculosis* Thymidylate Synthases, ThyA and ThyX

**DOI:** 10.1371/journal.pone.0002237

**Published:** 2008-05-21

**Authors:** Joshua H. Hunter, Ramesh Gujjar, Cullen K. T. Pang, Pradipsinh K. Rathod

**Affiliations:** 1 Department of Chemistry, University of Washington, Seattle, Washington, United States of America; 2 Department of Global Health, University of Washington, Seattle, Washington, United States of America; University of Arkansas, United States of America

## Abstract

**Background:**

*Mycobacterium tuberculosis* kills approximately 2 million people each year and presents an urgent need to identify new targets and new antitubercular drugs. Thymidylate synthase (TS) enzymes from other species offer good targets for drug development and the *M. tuberculosis* genome contains two putative TS enzymes, a conventional ThyA and a flavin-based ThyX. In *M. tuberculosis*, both TS enzymes have been implicated as essential for growth, either based on drug-resistance studies or genome-wide mutagenesis screens. To facilitate future small molecule inhibitors against these proteins, a detailed enzymatic characterization was necessary.

**Methodology/Principal Findings:**

After cloning, overexpression, and purification, the thymidylate-synthesizing ability of ThyA and ThyX gene products were directly confirmed by HPLC analysis of reaction products and substrate saturation kinetics were established. 5-Fluoro-2′-deoxyuridine 5′-monophosphate (FdUMP) was a potent inhibitor of both ThyA and ThyX, offering important clues to double-targeting strategies. In contrast, the folate-based 1843U89 was a potent inhibitor of ThyA but not ThyX suggesting that it should be possible to find ThyX-specific antifolates. A turnover-dependent kinetic assay, combined with the active-site titration approach of Ackermann and Potter, revealed that both *M. tuberculosis* enzymes had very low *k*_cat_ values. One possible explanation for the low catalytic activity of *M. tuberculosis* ThyX is that its true biological substrates remain to be identified. Alternatively, this slow-growing pathogen, with low demands for TMP, may have evolved to down-regulate TS activities by altering the turnover rate of individual enzyme molecules, perhaps to preserve total protein quantities for other purposes. In many organisms, TS is often used as a part of larger complexes of macromolecules that control replication and DNA repair.

**Conclusions/Significance:**

Thus, the present enzymatic characterization of ThyA and ThyX from *M. tuberculosis* provides a framework for future development of cell-active inhibitors and the biological roles of these TS enzymes in *M. tuberculosis*.

## Introduction

An estimated 2 million deaths occur annually due to infections of *Mycobacterium tuberculosis*, the causative agent of tuberculosis [1]. Multidrug-resistant tuberculosis, the recently discovered extensively drug-resistant form of the disease, and the numerous side effects of current drugs during the long course of drug treatment are ongoing problems [2]–[4]. Rifampicin, the newest first line antitubercular drug was introduced nearly 45 years ago [5]. Consequently there is an urgent need for identifying new drug targets and inhibitors that can effectively combat this disease.

**Figure 1:**
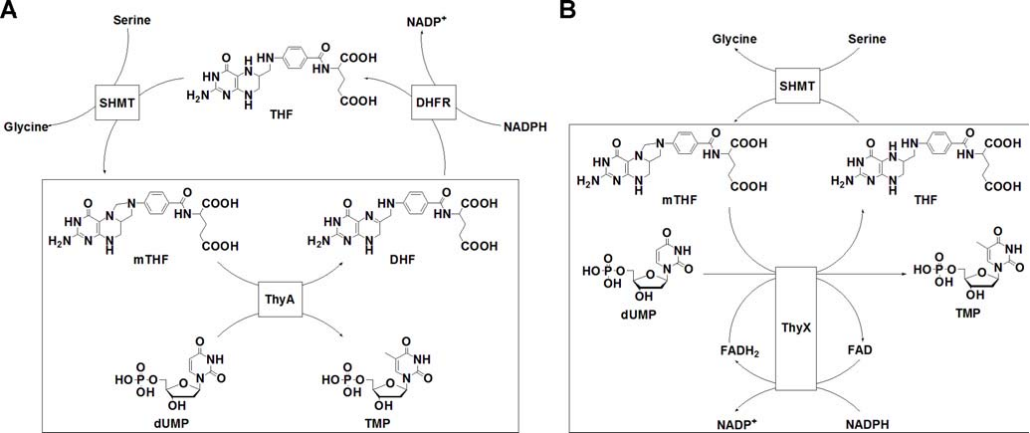
Different reaction complexities of ThyA and ThyX. (A) ThyA converts dUMP to TMP using mTHF as a cofactor. mTHF is regenerated by dihydrofolate reductase (DHFR) and serine hydroxymethyltransferase (SHMT). (B) ThyX converts dUMP to TMP using mTHF, NADPH, and FAD as cofactors. mTHF is regenerated by SHMT.

In other human diseases, the enzyme thymidylate synthase (ThyA) is an attractive target for drug development [6]. ThyA catalyzes the reductive methylation of 2′-deoxyuridine-5′-monophosphate (dUMP) to thymidine-5′-monophosphate (TMP) while utilizing *N* ^5^,*N* ^10^-methylene-5,6,7,8-tetrahydrofolate (mTHF) as the methyl donor and reductant in the reaction, yielding dihydrofolate (DHF) as a by-product (Fig. 1A) [7]. Even partial inhibition of ThyA causes an accumulation of 2′-deoxyuridine-5′-triphosphate (dUTP) and a cascade of events leading to “thymine-less” cell death [8], [9]. Many established and experimental anticancer drugs, such as 5-fluorouracil, Nolatrexed (AG337), Pemetrexed, and Raltitrexed (ZD1694), act through this enzyme [10]. Prior understanding of this general target in pharmacology, along with the vast number of potent lead inhibitors already available, should facilitate drug development against other indications including antibacterials [11]. It has recently been postulated that p-aminosalicylic acid, a commonly used antitubercular drug, involves *M. tuberculosis* ThyA, suggesting that inhibition of ThyA activity may be detrimental to *M. tuberculosis* growth and survival [12]. The *M. tuberculosis* genome sequence also lacks thymidine kinase, underscoring the essentiality of *de novo* TMP synthesis in this pathogen [13]. On this basis, selective inhibition of *M. tuberculosis* growth by ThyA inhibitors might not even require inhibitors selective for *M. tuberculosis* ThyA, as shown for the experimental treatment of malaria by inhibition of ThyA [14], [15].

In addition to the conventional ThyA, *M. tuberculosis* also appears to carry a flavin-dependent thymidylate synthase (FDTS or ThyX) [16]. Based on sequence and structural similarities to related enzymes in *Helicobacter pylori* and *Thermotoga maritima*, *M. tuberculosis* ThyX is expected to catalyze the reductive methylation of dUMP to afford TMP using mTHF as the methyl donor in the reaction (Fig. 1B) [13], [16], [17]. In other species, ThyX utilizes NADPH and a bound FAD chromophore as the reductant for the reaction. Indeed, crystal structure and sequence analysis have found that *M. tuberculosis* ThyX binds NADP^+^ and FAD and has no direct similarity to ThyA [13]. In addition, site directed mutagenesis studies have identified several amino acid residues, including a conserved “ThyX motif” that are necessary for *M. tuberculosis* ThyX tritium-release activity [18]. Direct confirmation of full thymidylate synthase activity and its kinetic properties are still necessary because of the low sequence homology of the ThyX family of enzymes across species [13]. There is pharmacological interest in this enzyme because the *thyX* gene is absent in humans and transposon site hybridization (TraSH) experiments indicate that *Mycobacterium thyX* is an essential gene for optimal growth of the pathogen [19], [20].

Despite the genomic, crystallographic, and mutagenesis studies, many of the fundamental enzymatic properties, such as the substrate and inhibitor binding abilities, of *M. tuberculosis* ThyA and ThyX remain unknown. Understanding the biochemistry of these enzymes is a necessary foundation needed for prioritizing drug development strategies and assigning biological function with confidence. In this study, we report on the expression, purification, kinetic properties, and inhibitor-binding preferences of both *M. tuberculosis* ThyA and ThyX. While the kinetic constants for substrate binding are not unusual, both enzymes had surprisingly low turnover rates. This evolutionary strategy raises important questions about possible alternate or additional roles of these enzymes in microbial functions. Regarding drug development, given that individual cell types may be specifically targeted using selective transport and selective drug activation properties of the cell, our initial results suggest that fluorinated pyrimidines may be used to inhibit both thymidylate synthase enzymes simultaneously. In contrast different folate analogues may be customized to selectively inhibit one enzyme over another.

## Results and Discussion

### Expression and purification

**Figure 2:**
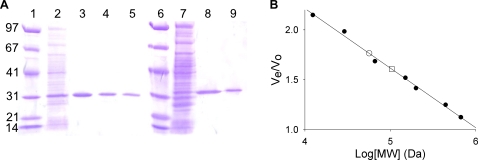
Analysis of *M. tuberculosis* ThyA and ThyX by SDS-PAGE and size exclusion chromatography. (A) Purification of ThyA and ThyX analyzed by SDS-PAGE (Lanes: 1, Molecular weight markers (see materials and methods); 2, 15 µg soluble lysate fraction; 3, 2 µg *M. tuberculosis* ThyA after Ni^2+^ column; 4, 2 µg *M. tuberculosis* ThyA after Q-sepharose column; 5, 2 µg *M. tuberculosis* ThyA after size exclusion; 6, Same as 1; 7, 15 µg soluble lysate fraction from ThyX expression; 8, 2 µg *M. tuberculosis* ThyX after Ni^2+^ column; 9, 2 µg *M. tuberculosis* ThyX after Q-sepharose column. (B) Analysis of ThyA and ThyX by size exclusion chromatography. Protein standards (•) eluted at 47.79, 53.74, 59.69, 67.64, 72.62, 80.56, 94.80, and 102.65 mL (see materials and methods). ThyA (○) eluted at 84.35 mL. ThyX (□) eluted at 76.83 mL.

Histidine-tagged *M. tuberculosis* ThyA was overexpressed from BL21(DE3) pLysS *E. coli*. Purification by nickel affinity, ion-exchange, and size exclusion chromatography afforded pure protein (Fig. 2A). A total of 18.5 mg of pure protein was obtained from 1 L of bacterial culture. Size exclusion chromatography (Fig. 2B) suggested that the protein had an apparent mass of 57 kDa, which is within 10% of the predicted mass of a dimer (61.4 kDa). This is the first purification of ThyA from *Mycobacteria*. Similarly, histidine-tagged *M. tuberculosis* ThyX was overexpressed from BL21(DE3) pLysS *E. coli*. Again, purification by nickel affinity and ion-exchange chromatography afforded pure protein (Fig. 2A). A total of 8 mg of pure protein was obtained from 1 L of bacterial culture (more recent protein preparations have routinely yielded 20 mg of ThyX per liter of culture). Size exclusion chromatography (Fig. 2B) suggested that this protein had an apparent mass of 104 kDa, which is within 10% of the predicted mass of a tetramer (114.8 kDa). This agrees with the previously reported crystal structure, which shows that the enzyme exists as a tetramer when crystallized [16].

### Lack of autologous RNA binding

**Figure 3:**
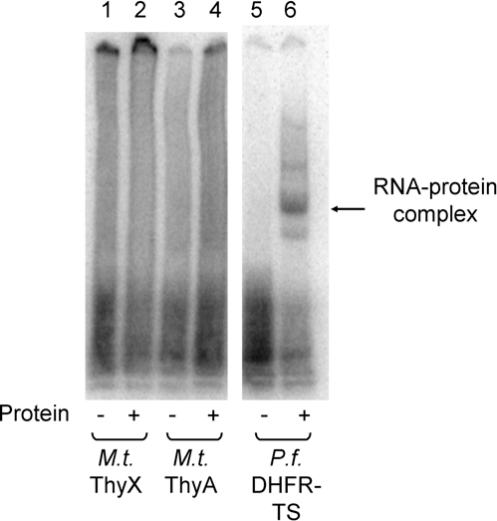
RNA binding by *M. tuberculosis* ThyA and ThyX. *M. tuberculosis* thymidylate synthases do not bind to their cognate mRNA. 0.35 nM of ^32^P labeled *M. tuberculosis* ThyX mRNA (lanes 1 and 2), *M. tuberculosis* ThyA mRNA (lanes 3 and 4), or *P. falciparum* DHFR-TS mRNA (lanes 5 and 6) was incubated in the presence or absence (lanes 1, 3, and 5) of purified *M. tuberculosis* ThyX (3.5 µM, lane 2), *M. tuberculosis* ThyA (3.5 µM, lane 4), or *P. falciparum* DHFR-TS (500 nM, lane 6). Unbound RNA was digested with 3 units of RNase T1 prior to electrophoresis [22].

Autologous RNA binding by the classical thymidylate synthase, ThyA, can play an important role in pharmacology. Whether a protein binds its own RNA, and whether the resulting autologous translational inhibition can be reversed with enzyme inhibitors, can be important contributing variables to species-specific drug action [21], [22]. Human and *P. falciparum* ThyA have been shown to bind their cognate mRNA coding sequence and to inhibit their own translation [21]–[23]. The mRNA binding abilities of *M. tuberculosis* ThyA and ThyX were probed using a gel-shift assay (Fig. 3). Neither ThyA nor ThyX were found to bind their own mRNA. These initial results suggest that the expression levels of *M. tuberculosis* ThyA and ThyX are not regulated by classic autologous translational feedback loops and instead may be dominated by regulation of RNA transcription.

### Activity and substrate binding

**Figure 4:**
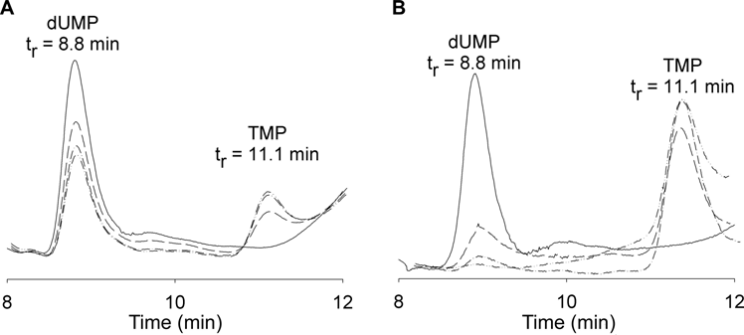
HPLC analysis of *M. tuberculosis* ThyA and ThyX products. (A) *M. tuberculosis* ThyA reaction products. Based on absorbance at 260 nm, the peak corresponding to dUMP (t_r_ = 8.8 min) decreases while the peak corresponding to TMP (t_r_ = 11.1 min) increases over a 120 minute time course (0, 30, 60, and 120 minute time points). (B) *M. tuberculosis* ThyX reaction products. The peak corresponding to dUMP (t_r_ = 8.8 min) decreases while the peak corresponding to TMP (t_r_ = 11.1 min) increases over a 120 minute time course (0, 30, 60, and 120 minute time points).

The thymidylate-synthesizing abilities of *M. tuberculosis* ThyA and ThyX were confirmed by reversed phase HPLC (Fig. 4). The products of the enzymatic reactions were loaded onto a C18 column and decreases in dUMP and increases in TMP were confirmed using known standards. This simple experiment was necessary, particularly for ThyX. *ThyA* genes are very highly conserved across species [11]. *M. tuberculosis* ThyA and the well studied human ThyA (PDB # 1HZW) have a 53% sequence identity, including several essential catalytic residues [7], [24], thus creating high confidence that *M. tuberculosis* ThyA would indeed catalyze synthesis of thymidylate. In contrast, while the *M. tuberculosis thyX* gene product contains certain sequence motifs and structural elements identifying it as a TS [16], overall homology between ThyX sequences is low [13] and there was no direct proof of its ability to synthesize thymidylate.

**Figure 5:**
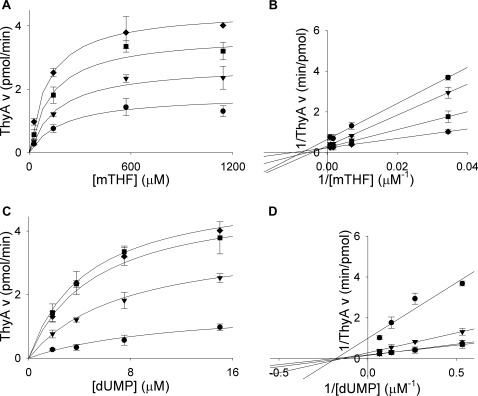
Steady-state kinetics of *M. tuberculosis* ThyA. (A) Titrations of mTHF (29, 143, 571, 1143 µM) with varying concentrations of dUMP (•, 1.875 µM; ▾, 3.75 µM; ▪, 7.5 µM; ♦, 15 µM). (B) Double reciprocal plot of data from plot A showing a sequential reaction mechanism. (C) Titrations of dUMP (1.875 µM, 3.75 µM, 7.5 µM, 15 µM) with varying concentrations of mTHF (•, 29 µM; ▾, 143 µM; ▪, 571 µM; ♦, 1143 µM). (D) Double reciprocal plot of data from plot C showing a sequential reaction mechanism. Lines in A and C are plots of Michaelis-Menten equations with calculated constants. Lines in B and D are linear regressions. Error bars are propagation of standard deviations.

**Figure 6:**
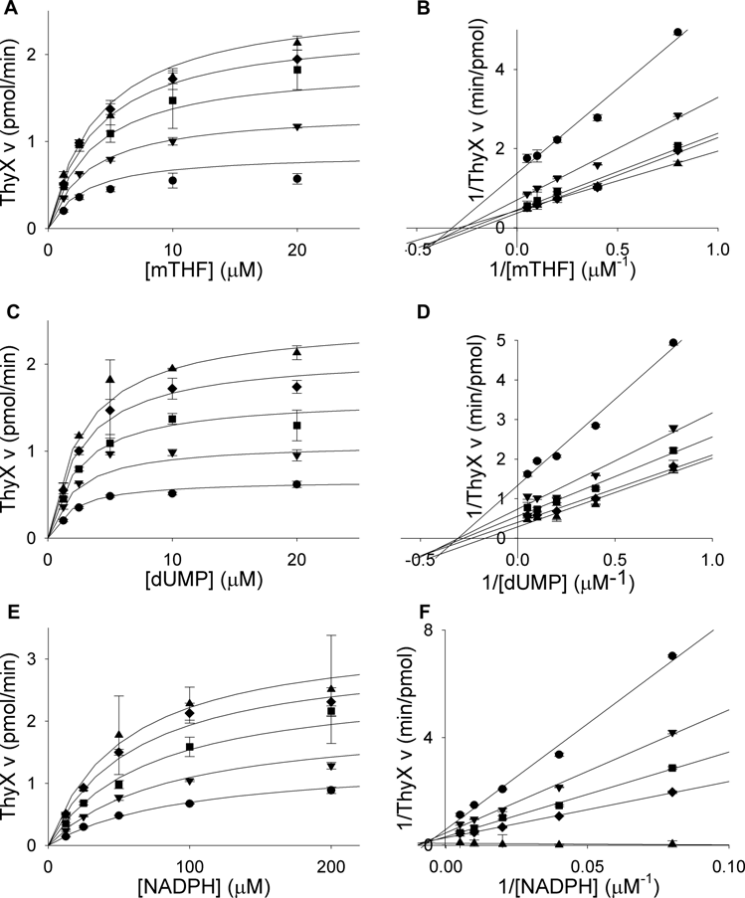
Steady-state kinetics of *M. tuberculosis* ThyX. (A) Titrations of mTHF (1.25, 2.5, 5, 10, 20 µM) with varying concentrations of dUMP (•, 1.25 µM; ▾, 2.5 µM; ▪, 5 µM; ♦, 10 µM; ▴, 20 µM). (B) Double reciprocal plot of data from plot A showing a sequential reaction mechanism. (C) Titrations of dUMP (1.25, 2.5, 5, 10, 20 µM) with varying concentrations of mTHF (•, 1.25 µM; ▾, 2.5 µM; ▪, 5 µM; ♦, 10 µM; ▴, 20 µM). (D) Double reciprocal plot of data from plot C showing a sequential reaction mechanism. (E) Titrations of NADPH (12.5, 25, 50, 100, 200 µM) with varying concentrations of mTHF (•, 1.25 µM; ▾, 2.5 µM; ▪, 5 µM; ♦, 10 µM; ▴, 20 µM) in the presence of 2.86 µM dUMP. (F) Double reciprocal plot of data from plot E showing a sequential reaction mechanism. Lines in A, C, and E are plots of Michaelis-Menten equations with calculated constants. Lines in B, D, and F are linear regressions. Error bars are propagation of standard deviations.

Thymidylate synthase activities of the isolated proteins were also verified using a tritium-release assay which depends on deprotonation at the 5-position of the pyrimidine ring. ThyA and ThyX produced 210 nmol and 9 nmol of tritiated water per minute per mg of protein, respectively (Table 1). ThyX activity was also verified by NADPH oxidation assay (data not shown). To examine substrate binding, the purified enzymes were assayed with increasing concentrations of either dUMP or mTHF, with varying concentrations of the second substrate. Similarly, ThyX was assayed with NADPH, with varying dUMP or mTHF. The enzymes displayed standard Michaelis-Menten curves for all substrates tested (Fig. 5 and 6). Fitting of the data to the appropriate equation revealed *K*_m_ values of 4±0.7 µM for dUMP and 70±30 µM for mTHF for *M. tuberculosis* ThyA (Table 2). These values are similar to the experimentally determined values for human ThyA (1.8 or 2.5 µM for dUMP and 12 or 31 µM for mTHF) [25], [26]. The kinetic constants calculated for *M. tuberculosis* ThyX were 3±0.7 µM for dUMP, 4.0±1 µM for mTHF, and 47±10 µM for NADPH (Table 2). These values were also similar to experimentally determined values for other ThyX enzymes, 6–65 µM for dUMP, 20–24 µM for mTHF, and 26 µM–4 mM for NADPH [27]–[31].

**Table 1:** Purification of *M. tuberculosis* ThyA and ThyX.

Enzyme and Purification step	Total amount of protein (mg)	Total activity (nmol/min)	Sp. activity (nmol/min/mg)	Purification factor	Yield (%)
*M. tuberculosis* ThyA					
Cell lysate	490	18,620	38	1	100
Purified protein	18.5	3,885	210	5.5	21
*M. tuberculosis* ThyX					
Cell lysate	340	782	2.3	1	100
Purified protein	8	72	9	4	9

**Table 2:** Comparison of kinetics of Human ThyA to *M. tuberculosis* ThyA and ThyX.

	*K*_m_ (µM)				*K*_i_ (nM)	
Enzyme	dUMP	mTHF	NADPH	*k*_cat_ (min^−1^)	FdUMP	1843U89
*M. tuberculosis*						
ThyA	4±0.7	70±30	-	18	2±0.2	18±1.0
ThyX	3±0.7	4±1	47±10	0.4	100±10	11,200±900
Human						
ThyA	2.5^a^, 1.8^b^	12^a^, 31^b^	-	150^c^	1.7^b^	0.09^d^

a From Dev *et al.* [46].

b From Dolnick and Cheng [25].

c From Davisson *et al.* [26].

d From Pendergast *et al.* [44].

### Inhibitor binding

**Figure 7:**
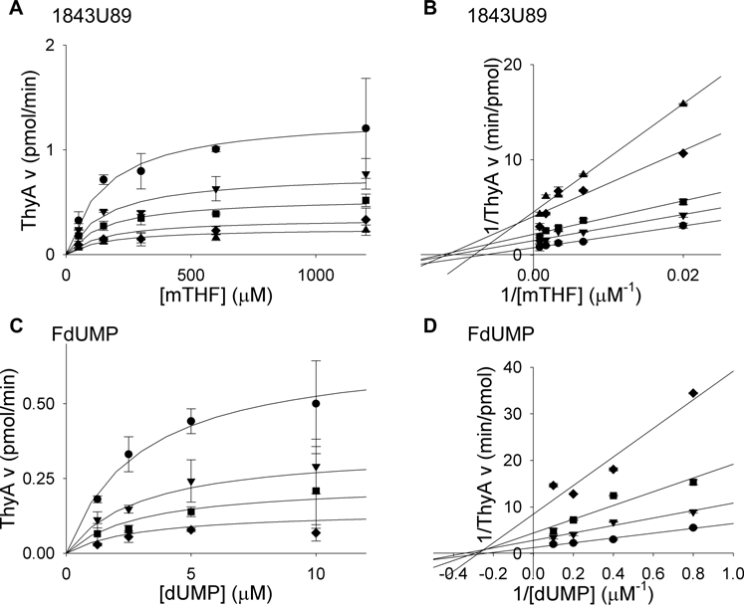
Inhibition of *M. tuberculosis* ThyA by 1843U89 and FdUMP. (A) Titrations of mTHF (50, 150, 300, 600, 1200 µM) with varying concentrations of 1843U89 (•, 0 nM; ▾, 12.5 nM; ▪, 25 nM; ♦, 50 nM; ▴, 75 nM) in the presence of 3.75 µM dUMP. (B) Double reciprocal plot of data from plot A showing a noncompetitive inhibition mechanism. (C) Titrations of dUMP (1.25, 2.5, 5, 10 µM) with varying concentrations of FdUMP (•, 0 nM; ▾, 2.5 nM; ▪, 5 nM; ♦, 10 nM) in the presence of 142.9 µM mTHF. (D) Double reciprocal plot of data from plot C showing a noncompetitive inhibition mechanism. Lines in A and C are plots of Michaelis-Menten equations with calculated constants. Lines in B and D are linear regressions. Error bars are propagation of standard deviations.

**Figure 8:**
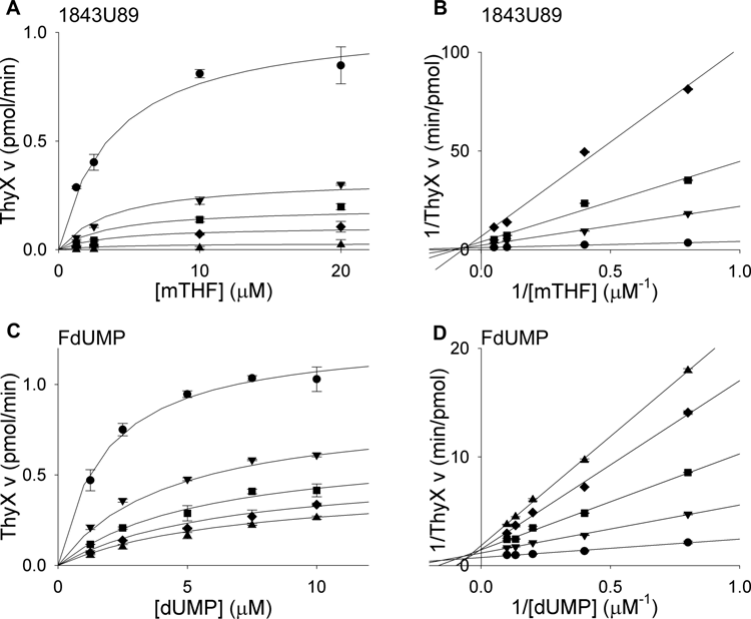
Inhibition of *M. tuberculosis* ThyX by 1843U89 and FdUMP. (A) Titrations of mTHF (1.25, 2.5, 10, 20 µM) with varying concentrations of 1843U89 (•, 0 µM; ▾, 25 µM; ▪, 50 µM; ♦, 100 µM; ▴, 400 µM) in the presence of 2.86 µM dUMP. (B) Double reciprocal plot of data from plot A showing a noncompetitive inhibition mechanism. (C) Titrations of dUMP (1.25, 2.5, 5, 7.5, 10 µM) with varying concentrations of FdUMP (•, 0 nM; ▾, 250 nM; ▪, 500 nM; ♦, 750 nM; ▴, 1000 nM) in the presence of 8 µM mTHF. (D) Double reciprocal plot of data from plot C showing a mixed inhibition mechanism. Lines in A and C are plots of Michaelis-Menten equations with calculated constants. Lines in B and D are linear regressions. Error bars are propagation of standard deviations.

Many folate and nucleotide analogs target TS enzymes [10]. One interesting challenge in *M. tuberculosis* pharmacology is to determine which compound class will inhibit which TS enzyme. Inhibition of *M. tuberculosis* ThyA and ThyX by the potent nucleotide-based inhibitor FdUMP, and the folate-based inhibitor 1843U89, were analyzed using the tritium-release assay. Based on similarity between substrates and inhibitors, FdUMP was analyzed with respect to dUMP, and 1843U89 was probed with respect to mTHF. Titrating the enzyme with increasing concentrations of substrate with various concentrations of the inhibitors revealed standard Michaelis-Menten curves (Fig. 7 and 8). Fitting of the data to the appropriate equation, based on double reciprocal plots, provided the *K*_i_ values.

FdUMP inhibited both ThyA and ThyX (Table 2). While this nucleotide-based inhibitor is a more potent inhibitor of ThyA (*K*_i_, 2 nM), it still shows mid-nanomolar level inhibition of ThyX (*K*_i_, 100 nM). This implies that fluorinated pyrimidine analogs could be used to inhibit both enzymes simultaneously. Again, specificity may be achieved from selection of appropriate fluorinated nucleotide, as is the case in experimental malaria chemotherapy [15], [32], [33].

While 1843U89 inhibited ThyA with a *K*_i_ of 18 nM, it displaying a 600-fold weaker binding to ThyX (Table 2). This compound was originally designed to inhibit human ThyA which is very similar in amino acid sequence to *M. tuberculosis* ThyA and quite dissimilar to *M. tuberculosis* ThyX. Just as 1843U89 is highly selective for ThyA over ThyX, basic thermodynamic principles suggest that reciprocal inhibitors for *M. tuberculosis* ThyX should exist. Such is the case in the selective inhibition of *P. falciparum* dihydroorotate dehydrogenase (DHODH) over human DHODH. Initially, it was shown that common DHODH inhibitors were approximately 2,000–5,000-fold selective for the human enzyme over the *P. falciparum enzyme* [34]. A subsequent high-throughput screen identified inhibitors up to 12,500-fold selective for *P. falciparum* DHODH over human DHODH [35], [36]. In addition to implications for future drug development strategies, TS-selective folate inhibitors should play an important role in untangling the biological roles of these two enzymes in *M. tuberculosis* biology.

### Low turnover

**Figure 9:**
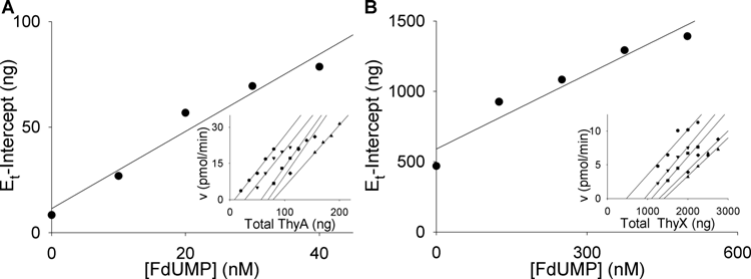
Ackermann-Potter plots of ThyA and ThyX activity. (A) Graph of x-intercepts from primary plot lines (inset) versus FdUMP concentration (0, 10, 20, 30, 40 nM). Inset plot: titrations of *M. tuberculosis* ThyA with varying concentrations of FdUMP in the presence of 1200 µM mTHF and 20 µM dUMP. •, 0 nM FdUMP (20, 35, 50, 65, 80 ng ThyA); ▾, 10 nM FdUMP (50, 65, 80, 95, 110 ng ThyA); ▪, 20 nM FdUMP (80, 95, 110, 125 ng ThyA); ♦, 30 nM FdUMP (110, 125, 140, 155 ng ThyA); ▴, 40 nM FdUMP (155, 170, 185, 200 ng ThyA). (B) Graph of x-intercepts from primary plot lines (inset) versus FdUMP concentration (0, 125, 250, 375, 500 nM). Inset plot: titrations of *M. tuberculosis* ThyX with varying concentrations of FdUMP in the presence of 20 µM mTHF, 20 µM dUMP, 1 mM NADPH, and 10 µM FAD. •, 0 nM FdUMP (1.25, 1.5, 1.75, 2, 2.25 µg ThyX); ▾, 125 nM FdUMP (1.25, 1.5, 1.75, 2 µg ThyX); ▪, 250 nM FdUMP (1.5, 1.75, 2, 2.25, 2.5 µg ThyX); ♦, 375 nM FdUMP (2, 2.25, 2.5, 2.75 µg ThyX); ▴, 500 nM FdUMP (2, 2.25, 2.5, 2.75 µg ThyX). Error bars were omitted to simplify the graphs.

The present kinetic experiments revealed that *M. tuberculosis* ThyA and ThyX have very low catalytic rates for the synthesis of TMP. Initially, V_max_ with known protein amounts in the reactions, we obtained apparent *k*_cat_ values of 6 min^−1^ and 0.25 min^−1^ for ThyA and ThyX respectively. To rule out potential artifacts due to misfolded or inactive protein, the method of Ackermann and Potter was applied to determine the absolute *k*_cat_ values of catalytically active ThyA and ThyX protein molecules [37]–[39]. Activity was determined with varying amounts of the purified enzymes titrated with increasing concentrations of FdUMP (Fig. 9).

The slopes from the primary and secondary Ackermann-Potter plots revealed a *k*_cat_ value of 18 min^−1^ for *M. tuberculosis* ThyA. This is still an order of magnitude lower than the values for human and *P. falciparum* ThyA (150 min^−1^ and 120 min^−1^, respectively [26], [40]). The rate of turnover by *M. tuberculosis* ThyX was even slower, with a *k*_cat_ value of 0.4 min^−1^. This too was determined using the Ackermann-Potter method. Of all the ThyX enzymes studied from other microorganisms, which show as much as 100-fold variations in specific activity for ThyX [27], [28], [30], [31], the *M. tuberculosis* ThyX enzyme is the slowest. The ThyA and ThyX turnover numbers raise some important biological questions relevant to TB pharmacology. Due to the low turnover rates of ThyX enzymes in general, several studies have questioned whether the true biological substrates for this enzyme have been identified [31], [41], [42]. While the substrates used in the present work were able to catalyze turnover *in vitro*, it is possible that they do not provide an accurate measure of *in vivo* activity. Alternatively, the low turnover rate may be related to an important evolutionary characteristic of *M. tuberculosis*. It is a very slow growing pathogen, presumably with limited needs for TMP. Normally, one would assume that such an organism would evolve to down-regulate thymidylate synthesizing protein expression, compared to rapidly proliferating cells. The present chemical data raises the possibility that this slow-growing pathogen may have evolved to decrease the catalytic activity of individual enzyme molecules, perhaps to preserve total protein quantities for other purposes. Cellular role of TS proteins can extend beyond generating TMP. ThyA is often found as a part of a larger complex of proteins and RNA that are involved in control of cell replication as well as DNA repair [43]. If ThyA levels and ThyX levels are not low, development of pathogen inhibitors will have to rely on selectivity mechanism other than low target levels.

### Concluding remarks

The present chemical studies help to set the initial foundation to further evaluate *M. tuberculosis* ThyA and ThyX as chemotherapeutic targets against tuberculosis. Even though *M. tuberculosis* carries two genes for thymidylate synthesis, both of which have been implicated as important in growth and survival of this major human pathogen, the biological data available to date do not define the detailed metabolic role and the vulnerability of *M. tuberculosis* to inhibitors of these enzymes. If detailed genetic manipulations of individual genes formally show that both genes are essential, chemical genetic approaches based on the present enzymatic studies will follow. These should facilitate an understanding of whether target inhibition of one or both enzymes is needed to kill *M. tuberculosis* in its growth phase, whether target inhibition of one or both enzymes is needed to kill *M. tuberculosis* in its latent phase, whether the bacteria can upregulate each of the proteins in the presence of inhibitors to overcome drug action, whether one gene can facilitate resistance to inhibitors directed at the other TS, and whether selective dual targeting may be required to successfully shut off TMP synthesis.

## Materials and Methods

### Reagents

FAD, NADPH, tetrahydrofolic acid, Pefabloc SC, DNAseI, FdUMP, dUMP, TMP and protein standards for size-exclusion chromatography were purchased from Sigma-Aldrich (St. Louis, MO). [5-^3^H]-dUMP was purchased from Moravek Biochemicals (Brea, CA). His GraviTrap columns, DEAE Sepharose Fast Flow, Q Sepharose Fast Flow, and Superdex 200 were purchased from GE Healthcare (Piscataway, NJ). Protein concentrations were measured using the Protein Assay kit from Bio-Rad Laboratories (Hercules, CA). Low-range protein molecular weight markers were obtained from Bio-Rad Laboratories (Hercules, CA) (phosphorylase b, 97,400; serum albumin, 66,200; ovalbumin, 45,000; carbonic anhydrase, 31,000; trypsin inhibitor, 21,500; lysozyme, 14,400). The T7 Riboprope *in vitro* transcription system was obtained from Promega (Madison, WI). RNasin and RNase T1 were purchased from Ambion (Foster City, CA). Uridine 5′-triphosphate [α-^32^P] was purchased from American Radiolabeled Chemicals (St. Louis, MO). To prepare a 20 mM stock solution of methylene tetrahydrofolate (mTHF), solid tetrahydrofolic acid was dissolved in a buffer containing 125 mM TES, 60 mM MgCl_2_, 2.5 mM EDTA (pH 8.0), 190 mM 2-mercaptoethanol, and 16 mM formaldehyde. All, more dilute, mTHF solutions were made from this stock solution. mTHF solutions were stored under argon at −20°C. 1843U89 was synthesized as previously reported [44].

### Expression and purification of *M. tuberculosis* ThyA

The *thyA* gene was cloned from a pET24d(+) vector with a C-terminal His-Tag into a pET23d(+) vector with an N-terminal His-Tag using the forward primer (TTA CCA GCT AGC CTC GAG ATG CAC CAC CAC CAC CAC CAC ACG CCA TAC GAG GAC CTG CTG) and the reverse primer (GTT ATT GAG CTC CCC GGG TCA TAC CGC GAC TGG AGC TTT GAT C) including NheI and SacI restriction sites (underlined) and the plasmid construct was confirmed by sequencing. The protein was expressed in BL21(DE3) pLysS *E. coli* in 100 mL LB media containing 50 µg/mL ampicillin and 35 µg/mL chloramphenicol at 37°C overnight. About 50 mL of the overnight culture was added to 1 L of LB with the appropriate antibiotics. The 1 L culture was grown to an OD_600 nm_ of 0.6 and IPTG was added to give a final IPTG concentration of 1 mM. After 6 hours, the cells were harvested by centrifugation at 10,000×g at 4°C and the cells were resuspended, on ice, in 20 mM sodium phosphate (pH 7.4), 500 mM sodium chloride, 15 mM magnesium chloride, 20 mM imidazole, 20 µg/mL DNAseI, 1 mM Pefabloc SC, and 0.2 mg/mL lysozyme and lysed by sonication. Cell debris was removed by centrifugation at 10,000×g at 4°C and the cleared lysate was purified on a His GraviTrap column as specified by the manufacturer. The crudely purified protein was dialyzed against 75 mM potassium phosphate (pH 7.8), 0.1 mM EDTA, and 1 mM DTT and bound to a DEAE Sepharose Fast Flow column. The protein was eluted with a 0–250 mM NaCl gradient. Fractions containing ThyA were pooled, concentrated, and purified on a Superdex 200 size exclusion column with elution by 20 mM Tris (pH 7.8), 150 mM KCl, 1 mM DTT, and 0.1 mM EDTA. The enzyme was frozen at −20°C in the same buffer containing 20% glycerol.

### Expression and purification of *M. tuberculosis* ThyX

The gene for *M. tuberculosis thyX* with a C-terminal His-Tag was expressed as above [16]. ThyX bound the Ni^2+^ column tightly and was eluted with 20 mM sodium phosphate (pH 7.4), 1M NaCl, and 1M imidazole. The crudely purified protein was dialyzed, against 10 mM Tris HCl (pH 7.2), 50 mM NaCl, and 2 mM DTT, and bound to a Q Sepharose Fast Flow column. The protein was eluted with a 50–500 mM NaCl gradient. Fractions containing ThyX were pooled, dialyzed against 50 mM Tris HCl (pH 7.5), 200 mM NaCl, and 10% glycerol, and frozen at −20°C.

### Protein size

The approximate masses of ThyA and ThyX, in solution, were determined using size-exclusion chromatography. A mixture of protein standards consisting of: Blue Dextran (void volume marker), thyroglobulin (669 kDa), apoferritin (443 kDa), b-amylase (200 kDa), alcohol dehydrogenase (150 kDa), bovine serum albumin (66 kDa), carbonic anhydrase (29 kDa), and cytochrome C (12.4 kDa), was loaded onto a Superdex 200 size-exclusion column with a bed volume of 120 mL (the concentration of the standards was 1–2 mg/mL for each protein). The standards were eluted with 20 mM potassium phosphate (pH 7.8), 150 mM KCl, 1 mM DTT, and 0.1 mM EDTA with a flow rate of 0.7 mL/min. Samples of ThyA and ThyX were loaded onto the column at a concentration of approximately 1.5 mg/mL and eluted with the same buffer and flow rate. A plot of elution volume/void volume versus log of the molecular weight standards gave a linear data set whose regression line was used to calculate thymidylate synthase molecular weight from enzyme elution volumes [45].

### RNA binding assay

RNA binding experiments was measured as previously described [21], [22]. ThyA and ThyX mRNA was generated using the T7 Riboprobe *in vitro* transcription system (Promega) following the manufacturers protocol. The RNA was purified by phenol/chloroform extraction followed by ethanol precipitation. The amount of RNA transcribed was determined by measuring the OD_260 nm_ of the solution. Electrophoresis using 1% formamide agarose was used to determine the size and integrity of the RNA. Radiolabeled RNA was made by using 12 µM rUTP and adding 50 µCi of [α-^32^P] UTP. Labeled RNA was quantified by the ^32^P-incorporation assay following the protocol from Promega. RNA (0.35 nM labeled with 100,000 cpm ^32^P) was incubated with varying concentrations of purified enzyme in 10 mM HEPES pH 7.5, 3 mM MgCl_2_, 40 mM KCl, 5% glycerol, 200 mM 2-mercaptoethanol, and 1 unit of RNasin at 37°C for 15 minutes. The unbound RNA was digested by addition of 3 units of RNase T1 and incubation at 25°C for 10 minutes. Non-specific interactions were reduced by addition of 5 mg/ml heparin and incubation at 25°C for 10 minutes. The RNA-protein complexes were resolved on a 5% TBE polyacrylamide gel and visualized by autoradiography.

### TMP synthesis

Approximately 150 ng of *M. tuberculosis* ThyA, or 3 µg of *M. tuberculosis* ThyX, was added to a mixture of 60 µL mTHF (final mTHF concentrations were 143 µM for ThyA assay or 20 µM for ThyX assay), 15 µL of 140 µM dUMP, 3 µL of 350 µM FAD (for ThyX assay only), and 1 µL of 35 mM NADPH (for ThyX assay only) and water to give reaction volume of 105 µL. The reaction times varied from 30 to 120 minutes. To isolate the products, the reaction mixture was spun through a Microcon YM-10 centrifugal filter (MWCO = 10,000) at 13,000×g for 5 minutes. An additional 100 µL of water was added and the spin was repeated. The deproteinized reaction mixture was run on an HPLC using a Beckman reversed phase Ultrasphere ODS 5 µm C-18 column (4.6 mm ID, 150 mm length) with isocratic flow with 5 mM potassium phosphate (pH 7.0), 5 mM tetrabutylammonium dihydrogen phosphate, and 5% (v/v) acetonitrile, while monitoring elution at 260 nm. Standard solutions of dUMP, TMP, and mTHF were also run to assign the elution positions of reactants and products.

### Thymidylate synthase assay

In a standard tritium-release assay, 50 ng of *M. tuberculosis* ThyA or 1 µg of *M. tuberculosis* ThyX, in 1 µL, was added to 20 µL mTHF (mTHF concentrations in the reactions ranged from 5.7 µM–1143 µM for ThyA assay or 1.25 µM–20 µM for ThyX assay), 1 µL of 200 µM FAD (for ThyX assay only), and 1 µL of 2 mM NADPH (for ThyX assay only). The total reaction volume was brought to 30 µL with the addition of water. To initiate the reaction, 5 µL of 13.1 µM–140 µM [5-^3^H]-dUMP (0.4 Ci/mmol) was added. After a 25 minute reaction period, at room temperature, the reaction was terminated by the addition of 20 µL of a stop solution (a 3∶1 ratio of 2 N TCA∶4.3 mM dUMP). To remove unreacted substrates, 200 µL 10% (w/v) activated charcoal in water was added to the reaction mixture. The reaction tubes were incubated on ice for 15 minutes then spun at 13,000×g at 4°C for 10 minutes. A 100 µL aliquot of the supernatant was assayed by liquid scintillation counting to determine the amount of tritium-containing water produced by the reaction. Specific activities and V_max_ values were determined in the presence of saturating concentrations of substrates.

### Kinetic properties

The tritium release, at fixed time points, revealed initial reaction velocities needed to calculate the *K*_m_ values of dUMP, mTHF, and NADPH and the *k*_cat_ values of thymidylate synthase activity. See the legends for Fig. 5, 6, and 9 for substrate concentrations. Plots of reaction velocity (v) versus substrate concentration (A and/or B) were fit, using SigmaPlot 9.0, to the Michaelis-Menten equation for a sequential mechanism: v = (V_max_[A][B])/([A][B]+*K*_m_^A^[B]+*K*_m_^B^[A]+*K*_m_^B^ *K*_s_^A^). *k*_cat_ values were determined from Ackermann-Potter plots using the equation, Slope = (*k*_cat_[S])/([S]+*K*_m_) [37]–[39]. See the legend for Fig. 9 for enzyme and inhibitor concentrations.

### Inhibitory constants

The tritium-release assay was also used to determine the *K*_i_ values of the inhibitors for the ThyA and ThyX enzymes. Inhibitor and corresponding substrate concentrations were varied as listed in the legends of Fig. 7 and 8 (dUMP was varied against changing FdUMP in the reactions and mTHF was varied against changing 1843U89 in the reactions). The data yielded double reciprocal plots that were fit to linear regression lines. Plots of reaction velocity versus substrate concentration were fit, using SigmaPlot 9.0, to the appropriate Michaelis-Menten equation for an inhibition by a non-competitive: v = (V_max_[B])/((1+[I]/*K*_i_)(*K*_m_^B^+[B])), competitive: v = (V_max_[B])/(*K*_m_^B^(1+[I]/*K*_i_+[B]), or mixed inhibitor: v = (V_max_[B])/(*K*_s_^A^(1+[I]/*K*_i_)+[B](1+[I]/*K*_is_)).
